# Acoustically Driven Cortical δ Oscillations Underpin Prosodic Chunking

**DOI:** 10.1523/ENEURO.0562-20.2021

**Published:** 2021-07-09

**Authors:** J. M. Rimmele, D. Poeppel, O. Ghitza

**Affiliations:** 1 Department of Neuroscience, Max Planck Institute for Empirical Aesthetics, Frankfurt am Main 60322, Germany; 2 Max Planck NYU Center for Language, Music, and Emotion, Frankfurt am Main, Germany, New York, NY; 3 Department of Psychology and Center for Neural Science, New York University, New York, NY 10003; 4 Department of Biomedical Engineering and Hearing Research Center, Boston University, Boston, MA 02215

**Keywords:** auditory, δ-band, oscillations, prosodic chunking

## Abstract

Oscillation-based models of speech perception postulate a cortical computational principle by which decoding is performed within a window structure derived by a segmentation process. Segmentation of syllable-size chunks is realized by a θ oscillator. We provide evidence for an analogous role of a δ oscillator in the segmentation of phrase-sized chunks. We recorded magnetoencephalography (MEG) in humans, while participants performed a target identification task. Random-digit strings, with phrase-long chunks of two digits, were presented at chunk rates of 1.8 or 2.6 Hz, inside or outside the δ frequency band (defined here to be 0.5–2 Hz). Strong periodicities were elicited by chunk rates inside of δ in superior, middle temporal areas and speech-motor integration areas. Periodicities were diminished or absent for chunk rates outside δ, in line with behavioral performance. Our findings show that prosodic chunking of phrase-sized acoustic segments is correlated with acoustic-driven δ oscillations, expressing anatomically specific patterns of neuronal periodicities.

## Significance Statement

Oscillation-based models of speech perception postulate a cortical computational principle by which decoding is performed within a time-varying window structure, synchronized with the input on multiple time scales. At prelexical level, cycles of a flexible θ oscillator, locked to the input syllabic rhythm, constitute the syllabic windows. We find that the presence of cortical δ oscillations correlates with whether or not an input phrase-sized chunk rate is inside the δ range. This suggests that at the phrase time scale, a δ oscillator could play a role analogous to that of the θ oscillator at the syllable level. The segmentation process is realized by a flexible δ oscillator locked to the input rhythm, with δ cycles constituting phrase-sized windows.

## Introduction

Naturally spoken language is a stream of connected sounds, and although the speech acoustics contain no cues regarding the beginning or end of linguistic units a combination of interleaved cues (e.g., pauses, intonation, syllabic stress) are embedded in the acoustic stream. Information, broadly termed “accentuation” (e.g., intonation, stress, pauses), is used by listeners to indicate boundaries associated with linguistic units (Aubanel et al., 2016; Oganian and Chang, 2019). The process by which the input signal is partitioned into temporal segments to be linked to a variety of linguistic levels of abstraction (ranging from phonetic segments to syllables to words and, ultimately, prosodic and intonational phrases) is called “segmentation.”

The segmentation process has been shown to operate on time intervals associated with syllables (up to ∼250 ms; Brungart et al., 2007; Ghitza and Greenberg, 2009; Doelling et al., 2014; Kösem et al., 2018), and a similar process has been suggested to operate on the phrasal level (0.5–2 s; Ding et al., 2016; Ghitza, 2017; Martin and Doumas, 2017; Keitel et al., 2018). At the syllabic level, perceptual segmentation, or chunking, is by and large a prelexical process. Oscillation-based models propose that this segmentation is realized by flexible θ oscillations aligning their phase to the input syllabic rhythm (“speech tracking”), where the θ cycles mark the speech chunks to be decoded (Poeppel, 2003; Lakatos et al., 2005, 2019; Ahissar and Ahissar, 2005; Ding and Simon, 2009; Ghitza, 2011; Giraud and Poeppel, 2012; Peelle and Davis, 2012; Gross et al., 2013; Haegens and Zion Golumbic, 2018; Rimmele et al., 2018a; Assaneo et al., 2020; Hovsepyan et al., 2020; Pittman-Polletta et al., 2021). At the phrase level, phrase rhythm can affect segmentation (Gee and Grosjean, 1983; Martin, 2015; Deniz and Fodor, 2019; Hilton and Goldwater, 2020). There have been various studies aiming to quantify phrase length and rhythmicity (Clifton et al., 2006; Breen, 2018; Deniz and Fodor, 2019), suggesting that typical intonational phrases are about 1 s in duration (Auer et al., 1999; Inbar et al., 2020; Stehwien and Meyer, 2021). More specifically, the duration of intonational phrases spans a range between ∼0.5 and 1 s in English (slightly faster in some other languages; Inbar et al., 2020; Stehwien and Meyer, 2021). Prosodic segmentation (here also termed “prosodic chunking”) is based on intonation units that contain specific prosodic cues (such as pauses or pitch contour), which can pace the information flow at the phrasal time scale (Shattuck-Hufnagel and Turk, 1996; Inbar et al., 2020). The extent to which phrase level rhythmic structure supports segmentation and structural parsing was not widely studied. Here, we investigate the neural processing of rhythmic structure at the phrasal scale by analyzing how individuals’ group single digits into “phrase-sized” digit chunks. What kind of neuronal mechanism can realize this chunking process?

Cortical δ oscillators, with a frequency range (∼0.5–2 Hz) that corresponds to the phrasal time scale, were shown to be elicited during phrasal processing of speech or chunking processes at the phrasal scale (Buiatti et al., 2009; Ding et al., 2016; Bonhage et al., 2017; Meyer et al., 2017; Keitel et al., 2018; Boucher et al., 2019). δ Was observed in the posterior superior temporal, the inferior frontal, precentral, and temporal-parietal cortex using ECoG (Ding et al., 2016), and using EEG at bilateral middle and superior temporal areas (also fusiform gyrus; Bonhage et al., 2017) and at frontotemporal sites (Boucher et al., 2019). [Recall the ambiguous definition of the δ range in the literature, which covers a range of overlapping frequency bands inside the 0.5- to 4-Hz frequency range (Bonhage et al., 2017; Keitel et al., 2018; Bröhl and Kayser, 2020). Since we are interested in the segmentation of phrasal chunks, which in English are ∼0.5–1 s long (Miller, 1962; Inbar et al., 2020), we opted to define the δ frequency band to be 0.5–2 Hz.]

And behaviorally, it has been shown that performance is impaired when the chunk rate is outside of the δ range (Ghitza, 2017). These findings suggest a role of neuronal oscillatory mechanisms in the δ range in chunking at a phrasal time scale (see also Martin and Doumas, 2017; Ghitza, 2020). Little is known, however, about the brain areas that may recruit chunking-related δ oscillations.

**Figure 1.**
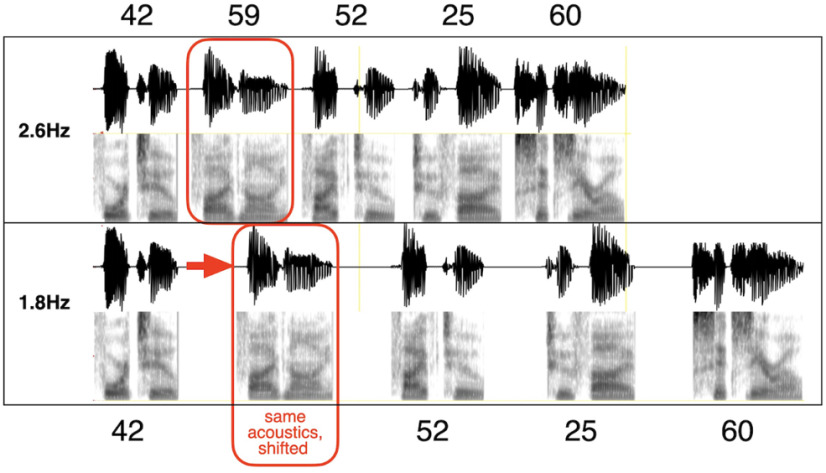
Chunk patterns and chunk rates for the 10-digit digit string 4259522560. The chunk pattern is 2222, with chunk rates of 1.8 Hz (inside δ) and 2.6 Hz (outside). Each chunk was synthesized as a two-digit unit, using the AT&T Text-to-Speech System accentuation (see text). Note that a particular two-digit chunk has the same acoustics, regardless of whether it occurs in the 1.8- or 2.6-Hz 2222 chunk condition (red box). The 1.8-Hz stimulus is generated by increasing the gap between the chunks (with identical chunk acoustics).

Here, we focus on the cortical mechanism that may be involved in acoustic-driven segmentation at a phrasal time scale, using sequences of digit chunks (with a minimal amount of content). We test the hypothesis that the decoding process is guided by a δ oscillator locked to the accentuation acoustic cues (Ghitza, 2017) by recording magnetoencephalography (MEG) data while participants performed a digit retrieval task. The digits in the string were grouped into chunks, with chunk rates either inside or outside to the δ frequency range (Fig. 1). The experiment addresses two questions: (1) Do elicited δ brain waves correlate with behavior, such that impaired performance in digit retrieval occurs if the chunk rate is outside of the δ range? (2) Where in the auditory pathway do those neuronal oscillations occur?

Our data show that in superior and middle temporal areas and in speech-motor planning and integration areas (IFG, PC, SMG), robust neural δ periodicities were elicited by chunk rates inside of the δ range but were diminished when the chunk rate was outside of the δ range, in line with behavioral performance. In speech-motor integration areas (SMG) and areas implicated in processing word form and meaning (middle temporal gyrus; MTG), periodicity was present albeit diminished even for chunk rates inside the δ range. The δ periodicities were acoustically driven, in sync with the input as long as the acoustic chunk rate was inside δ. δ Periodicities were diminished for chunk rates outside δ, although sufficient acoustic cues for chunking were present in all conditions. Thus, the failure to track the input-chunk-rate when it was outside of the δ range was not caused by insufficient acoustic cues but seems due to neuronal circuitry characteristics constraining the tracking of the chunks.

## Materials and Methods

### Participants

The data from 19 healthy right-handed (Oldfield, 1971) mean score: 75.22, SD: 18.08) participants were included in the study (mean age: 24.89 years, SD: 3.54; f = 14). Human subjects were recruited from the local community in Frankfurt. Two participants were excluded because of technical issues, and one participant because of outlier performance (i.e., performance < mean performance, 2 SD). Individual MRI scans were recorded for all except for two participants who did not fulfill the MRI scanning criteria. All participants gave written informed consent for participating in the study and received monetary compensation. The study was approved by the local ethics committee of the University Hospital Frankfurt (SG2-02-0128).

### Digit string stimuli

We used 10-digit long stimuli where we manipulated the pauses in-between digits according to the experimental conditions. We opted for digit sequences –material that is semantically unpredictable at the digit-chunk level (i.e., while semantic information is present at the single digit level, no semantic/contextual information is present at the digit-chunk level), to minimize the bottom-up/top-down interaction that is in play in setting perceptual boundaries for digit-chunks. The digit sequences were grouped into chunks, with a chunk pattern termed 2222. For example, the 2222 pattern of the sequence 4259522560 is [42 59 52 25 60]. Digits were presented as single digits, i.e., 42 was read as four-two and not as forty-two.

We used two chunk rates: 1.8 Hz (inside the δ range) and 2.6 Hz (at the outside border of the δ range, referred to as “outside”), termed conditions “1.8 Hz” and “2.6 Hz” (Fig. 1). Note that a third condition was used, which is not reported here. The condition was a “no-chunk” condition where digit chunks were presented at the rate of 2.6 Hz. However, besides top-down chunking information (provided by the instructions), there were no acoustic chunking cues. The neuronal findings resemble that of the 2.6-Hz chunking condition, confirming the main claims of this paper. They are reported elsewhere (Rimmele et al., 2020).

### Corpus

The text material comprised 100 10-digit long text strings. Stimuli were generated by using the AT&T Text-to-Speech System with the American English female speaker Crystal. [The AT&T-TTS system (http://www.wizzardsoftware.com/text-to-voice.php) uses a form of concatenative synthesis based on a unit-selection process, where the units are cut from a large, high-quality, prerecorded natural voice fragments. The system produces natural-sounding, highly intelligible spoken material with a realistic prosodic rhythm, with accentuation defined by the system-internal prosodic rules, and is considered to have some of the finest quality synthesis of any commercial product.] To generate stimuli with a 2222 chunk pattern, we first created a two-digit chunk vocabulary as follows. For each doublet of digits that exists in the 100 text strings, a naturally sounding two-digit chunk waveform was generated (naturalness was obtained by the AT&T system accentuation rules) resulting in a chunk-vocabulary. For a given text string, a 2222 10-digit stimulus was generated by concatenating the corresponding five two-digit chunk waveforms pulled from the chunk-vocabulary. The chunk rate was set by adjusting the gap duration in between two successive chunks, resulting in a stimulus with a temporal structure but without any contextual cues at the digit-chunk level. To enable the generation of stimuli with chunk rates greater than the δ frequency upper bound (at 2.6 Hz), the waveforms in all conditions were time compressed by a factor of 2.5, just below the auditory channel capacity (Ghitza, 2014). The duration of the 10-digit stimuli varied across conditions; for the 1.8-Hz condition: mean = 2.61 s (VAR = 85.6 ms), and for the 2.6-Hz condition: mean = 1.99 s (VAR = 85.6 ms).

For each of the 200 10-digit stimuli (100 stimuli for each of the 1.8- and 2.6-Hz conditions) a trial was created by concatenating the following waveform sequence: [one digit trial-count] [20-ms-long gap] [10-digit string] [500-ms-long gap] [two-digit target], resulting in one concatenated waveform per trial with durations that varied across trials and conditions. The 200 trials were scrambled, and the resulting pool of trials was divided into blocks, 50 trials per block. A jittered intertrial interval of 3–4.5 s was presented between trials. Overall, two different sets of stimuli were used.

### Task

Behavioral and MEG data were collected while participants performed a digit retrieval task, in the form of an adapted Sternberg target identification task (Sternberg, 1966; target ID task from here on): listeners heard a 10-digit stimulus followed by a two-digit-long target, and were asked to indicate whether or not the target was part of the utterance. A target position was always within a chunk. Note that the task is suitable for probing the role of acoustic segmentation in a memory retrieval task: a successful yes/no decision depends on how faithful the recognized chunk objects are, generated by a decoding process that, by hypothesis, depends on the goodness of segmentation.

### Procedure and paradigm

Participants were seated in front of a board for instructions in the MEG testing booth. Binaurally insert earplugs (E-A-RTONE Gold 3A Insert Earphones, Ulrich Keller Medizin-Technik) were used for stimulus presentation. Two button boxes (Current Designs) were used to record participants’ responses. The Psychophysics Toolbox (Brainard, 1997) was used to run the experiment. During the experiment, on each trial participants fixated the screen center (fixation cross) while listening to the digit sequences. The sounds were presented at a comfortable loudness level (∼70 dB SPL), which remained unchanged throughout the experiment. Overall, the experiment lasted ∼2.5 h, including preparation time, recording time, breaks, and postrecording questionnaires. Participants were presented with the task requirements. They were instructed that all sequences comprise concatenated chunks of two-digits. Before the experiment, all participants performed a short training of three trials (with feedback) to familiarize themselves with the stimuli and task. Participants were asked to indicate by button press (yes/no response; with the response hand balanced across participants; yes-hand right: *N* = 12) whether or not the target was part of the preceded utterance.

### MRI and MEG data acquisition

A 3 Tesla scanner (Siemens Magnetom Trio, Siemens) was used to record individual T1-weighted MRIs. MEG recordings were performed on a 269-channel whole-head MEG system (Omega 2000, CTF Systems Inc.) in a magnetically shielded booth. Data were acquired with a sampling rate of 1200 Hz, online denoising (higher-order gradiometer balancing) and online low pass filtering (cutoff: 300 Hz) was applied. Continuous tracking of the head position relative to the MEG sensors allowed correction of head displacement during the breaks and before each file saving of a participant, using the fieldtrip toolbox (http://fieldtrip.fcdonders.nl; Stolk et al., 2013).

### Behavioral analysis

A “yes–no” model for independent observations was used to compute dprime (Green and Swets, 1966). Four classes of response are considered: (1) hit: a “yes” response when the target chunk is present in the digit sequence; (2) correct rejection: a “no” response when the target chunk is absent; (3) miss: a “no” response when the target chunk is present; and (4) false alarm: a “yes” response when the target chunk is absent. Nonparametric Wilcoxon signed-rank tests (two-sided) were used to test differences in the mean dprime across conditions. The Bayes factor BF_10_ (Schönbrodt and Wagenmakers, 2018), which reflects the likelihood data arose from the alternative model, was computed using the software JASP (JASP Team, 2020; 10,000 samples) and default priors.

### MRI analysis

The FieldTrip toolbox (http://fieldtrip.fcdonders.nl; Oostenveld et al., 2011) was used for the MRI and MEG data analyses. The standard Montreal Neurologic Institute (MNI) template brain was used for participants where an individual MRI was missing. Probabilistic tissue maps (cerebrospinal fluid gray and white matter) were constructed from the individual MRIs. Next, a single shell volume conduction model (Nolte, 2003) was applied to retrieve the physical relation between sensors and sources. Between the individual T1 MRI and the MNI template T1 a linear warp transformation was computed. An 8-mm template grid, defined on the MNI template T1, was warped on the individual head space by inversely transforming it, based on the location of the coils during the MEG recording and the individual MRI. Next, based on the warped MNI grid and the probabilistic tissue map a forward model was computed, and applied for source reconstruction. This allowed aligning the grids of all participants to each other in MNI space for the across participants statistics.

### MEG preprocessing

Line-noise was removed using bandstop filters (49.5–50.5, 99.5–100.5, two-pass; filter order 4) and the data were bandpass filtered off-line (0.1–100 Hz, Butterworth filter; filter order 4). A common semi-automatic artifact detection procedure was applied: for artifact rejection, the signal was filtered to identify muscular artifacts (bandpass: 110–140 Hz) or jump artifacts (median filter) and z-normalized per time point and sensor. The *z* scores were averaged over sensors, to accumulate evidence for artifacts that occur across sensor. Trials that exceeded a predefined z value (muscular artifacts, z = 15; jumps, z = 30) were rejected. Trials were the range (min-max difference) in any sensor exceeded a threshold (threshold = 0.75e-5) were identified as containing slow artifacts and rejected. Down-sampling to 500 Hz was applied. The data were epoched (−3.5–5 s). Furthermore, when head movements exceeded a threshold (5 mm) a trial was rejected. Next, all blocks of recorded MEG data were concatenated. If high variance was detected at any sensor, the sensor was rejected. Finally, independent component analysis (infomax algorithm; Makeig et al., 1996) was used to remove eye-blink, eye-movement and heartbeat-related artifacts based on cumulative evidence from the component topography and time course.

### MEG source-level analysis

In a first step, the data were epoched (0–5 s). For the main analyses, only trials in which participants showed correct responses (i.e., hits and correct rejections) were selected. Next, the sensor-space measurements were projected and localized in source-space inside the brain volume (Van Veen et al., 1997) using linearly constrained minimum variance (LCMV) beamforming. A spatial filter was computed based on the individual leadfields for each participant and condition (λ = 10%; 0.8-cm grid). Next, all trials were epoched to the minimum stimulus duration in the corresponding condition (condition 1.8 Hz: 2.38 s; condition 2.6 Hz: 1.68 s).

### Cortical regions of interest (ROIs)

**Figure 2.**
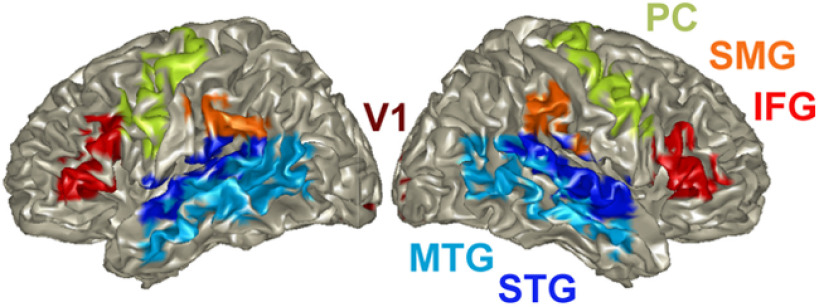
Cortical ROIs. The automated AAL (Tzourio-Mazoyer et al., 2002) was used to select Cortical regions of interest (ROIs) in left and right STG, middle temporal gyrus (MTG), and speech-motor planning and integration areas (IFG, PC, SMG). V1 was used as control region. ROIs are color coded.

The automated anatomic labeling atlas (AAL; Tzourio-Mazoyer et al., 2002) was used to select the ROIs as follows (Fig. 2):

- Superior temporal gyrus (STG; Temporal_sup_L/R): auditory association areas (Hickok and Poeppel, 2007; Binder et al., 2009)
- MTG (Temporal_Mid_L): implicated in processing word form and meaning
- IFG (Frontal_Inf_Tri_L/R): involved in speech-motor planning
- PC (Precentral_L/R), SMG (SupraMarginal_L/R): speech-motor integration
- Calcariane (Calcarine_L/R): primary visual cortex (as a control region)

We opted to omit Heschl’s gyrus (primary auditory cortex area) from the list of ROIs because of the very small number of voxels (three in the left, two in the right).

### Periodicity density function (PDF) within ROI

We aim to determine whether the elicited brain signal measured at any given voxel within a specific ROI shows periodicity, and if so, to extract the frequency. Ultimately, we seek to characterize the density function of the periodicities across all voxels in the ROIs of interest.

#### The aggregated cross-correlation measure (XCOV) of periodicity

To measure the neural response periodicity in individual voxels one could use one of several widely used measures, e.g., autocorrelation, where the first nontrivial peak indicates the period, or the intertrial phase coherence (ITPC), where the outcome would be the frequency distribution of the coherence function. Importantly, these measures build on the number of trials, M. The trial signals are noisy, both because of the SNR and because of the brain wave irregularity (which is why these methods average over trials). But what if M is too small? Here we used a newly proposed measure, termed XCOV, to measure periodicity across M trials. Broadly, we suggest taking advantage of the fact that, for M trials, we can generate about M^2^/2 cross-correlation functions. Recall that, unlike autocorrelation, the first peak of a cross-correlation function does not indicate the period but rather the delay between the two signals. Therefore, we run each of the M^2^/2 candidate pairs through a “match filter,” which determines whether the corresponding two signals have a “zero” delay. Such a pair will have a cross-correlation function similar to that of an autocorrelation function, i.e., its peak is at zero and its earliest nontrivial peak is at the period. Only the pairs that pass the test are cross-correlated and aggregated. Obviously, the number of cross-correlation functions qualified for aggregation is between M and M^2^/2, depending on how strict the match filter is. [For example, in the STG ROI, the mean number of trials over subjects for the “hit” response was M = 38, with a mean number of pairs of 703. The mean number of pairs that passed the test was 433 for the 1.8-Hz condition and 378 for the 2.6-Hz condition, about one degree of order bigger than M. A similar trend was observed for all ROIs.] We term the outcome of this measure as the XCOV function.

#### PDF within a ROI

**Figure 3.**
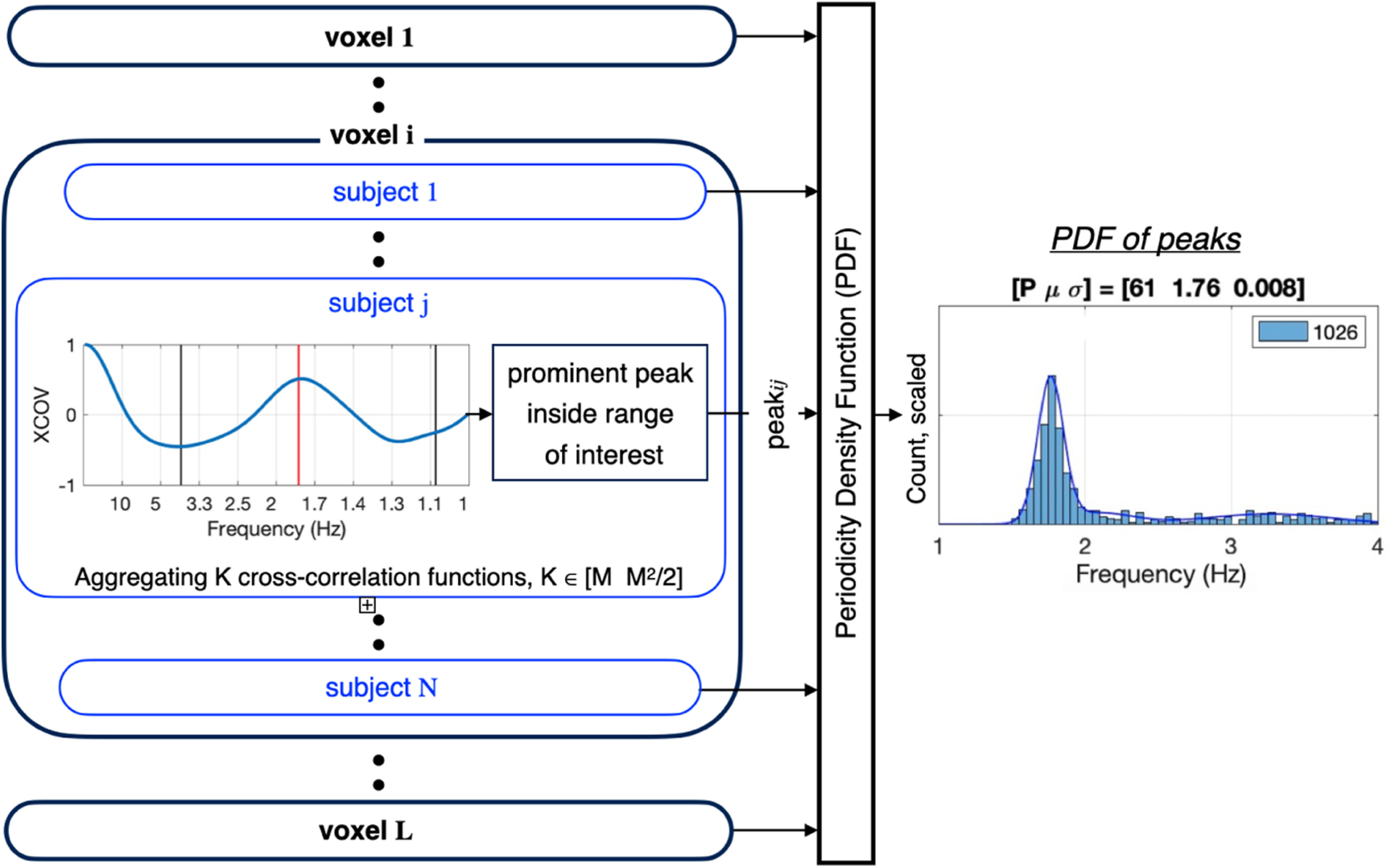
Analysis pipeline for deriving the PDF within a particular ROI. Shown is the resulting periodicity density function (PDF) for a given condition (say, the 1.8-Hz condition), a given response class (say, hit), and a given region of interest (ROI; say, STG). L voxels, N subjects, and M trials per subject are considered. For the i-th voxel and the j-th subject, periodicity is computed using a newly proposed measure method termed XCOV. First (data not shown), each brain signal is filtered to the frequency range of interest. Cross-correlations were computed using the filtered signals. Note that as a cross-correlation function, XCOV is computed against time-lags; the abscissa here shows the time-lag inverse, in frequency, hence going from right to left. The PDF is derived by (1) forming a histogram of the XCOV peak locations inside the frequency range of interest; (2) normalizing the histogram to L × N, the total number of data points; and (3) building a third order Gaussian Mixture Model (GMM) that fits the histogram. The GMM model is the desired PDF. The goodness of the PDF is quantified by in terms of *p* value, the percentage of datapoints inside the frequency range of interest with respect to the total number of datapoints (L × N); and the mean μ and variance σ of the prominent Gaussian component of a third order GMM. (The total number of data points is shown in the inset of each entry.)

Figure 3 details the analysis pipeline for deriving the PDF of the periodicities within a particular ROI. L voxels, N subjects, and M trials per subject are considered. First (data not shown), each brain signal is filtered to the frequency range of interest [low pass filter with cutoff frequency of 6 Hz for the (inside/outside) δ chunk rate analysis, and a bandpass filter with a [2–10] Hz frequency range, for θ (single digit rate) analysis]. [The filters were chosen with a bandwidth wider than the expected mean periodicities (1.8 and 2.6 Hz for δ, ∼4 Hz for θ), to let the XCOV analysis determine the periodicity PDFs without any bias.] Cross-correlations were computed using the filtered signals. Shown is the XCOV function at the i-th voxel, for the j-th subject, obtained by aggregating K cross-correlation functions. (Note that as a cross-correlation function, XCOV is computed against time-lags; the abscissa here shows the time-lag inverse, in frequency, hence going from right to left). The particular XCOV function shown in Figure 3 has a single peak at 1.76 Hz but note that, in general, an XCOV may have more than one local peak. Next, the location of the prominent peaks is extracted, with the number of prominent peaks as a parameter. (The prominence of a peak measures how much the peak stands out because of its intrinsic height and its location relative to other peaks in the range of interest.) In our analysis one prominent peak per XCOV is considered. Hence, for L voxels and N subjects, a maximum of L × N data points are available to construct a histogram, from which only those inside the frequency range of interest are used, and the resulting histogram is normalized to L × N. A third order Gaussian mixture model (GMM) that fits the histogram is the desired PDF. The “goodness” of the periodicity is quantified by in terms of P, the percentage of datapoints inside the frequency range of interest with respect to the total number of datapoints (L × N), and the mean μ and variance σ of the prominent Gaussian component of a third order GMM. (The total number of data points is shown in the inset of each entry.)

### Software accessibility statement

Analysis code will be made available on request.

## Results

### Behavioral results

**Figure 4.**
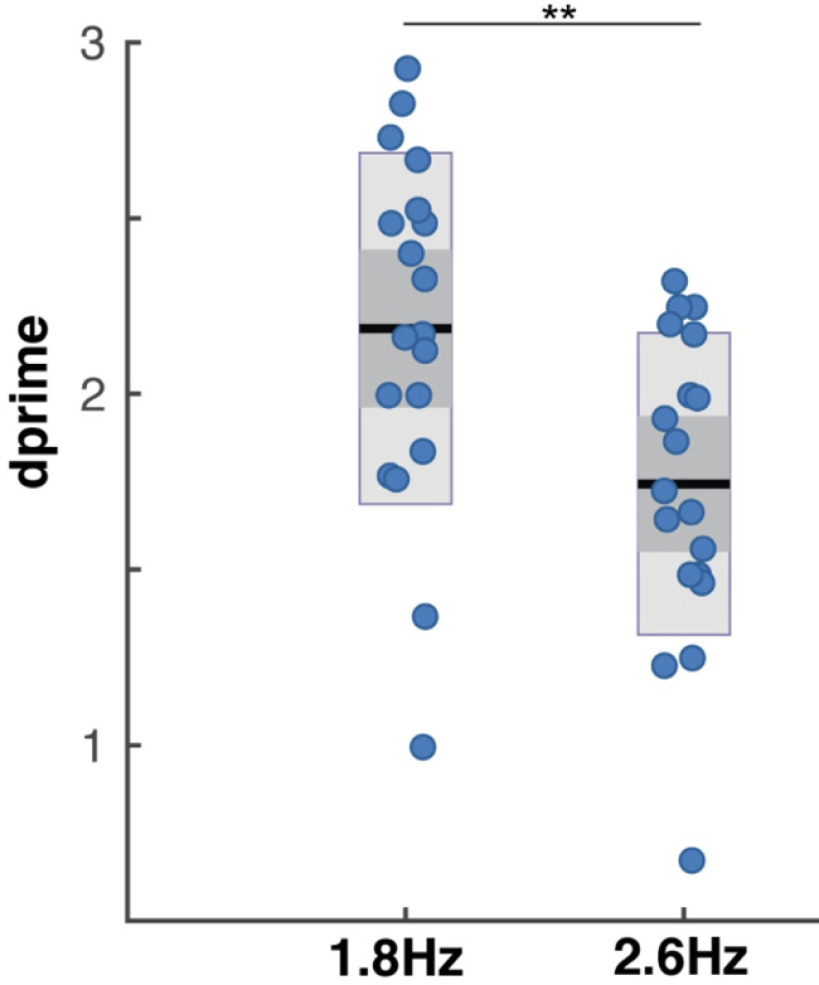
Behavioral performance in the digit retrieval task. Dprime values are displayed, as measure of performance accuracy, separately for each condition. Blue dots indicate individual dprime scores, black lines indicate the mean dprime scores, dark gray bars indicate the ±1 SEM and light gray bars the confidence interval. Significance is indicated by **(*p* < 0.01). The performance was higher in the 1.8-Hz acoustical chunk (inside δ chunking), compared with the 2.6-Hz acoustical chunk condition (outside of δ; replicating findings in Ghitza (2017)).

Dprime scores were the highest in the 1.8-Hz condition (mean = 2.19, SD = 0.4; Fig. 4), i.e., when the chunk rate is inside the δ frequency range. Lower dprime scores were registered in the 2.6-Hz condition (mean = 1.74, SD = 0.42), when the chunk rate was just at the outside edge of the δ range. The difference in scores was significant (1.8-Hz condition vs 2.6 Hz: *W* = 177, *p* < 0.001, *r* = 0.863; BF_10_ = 199.6).

### PDF of elicited brain waves

**Figure 5.**
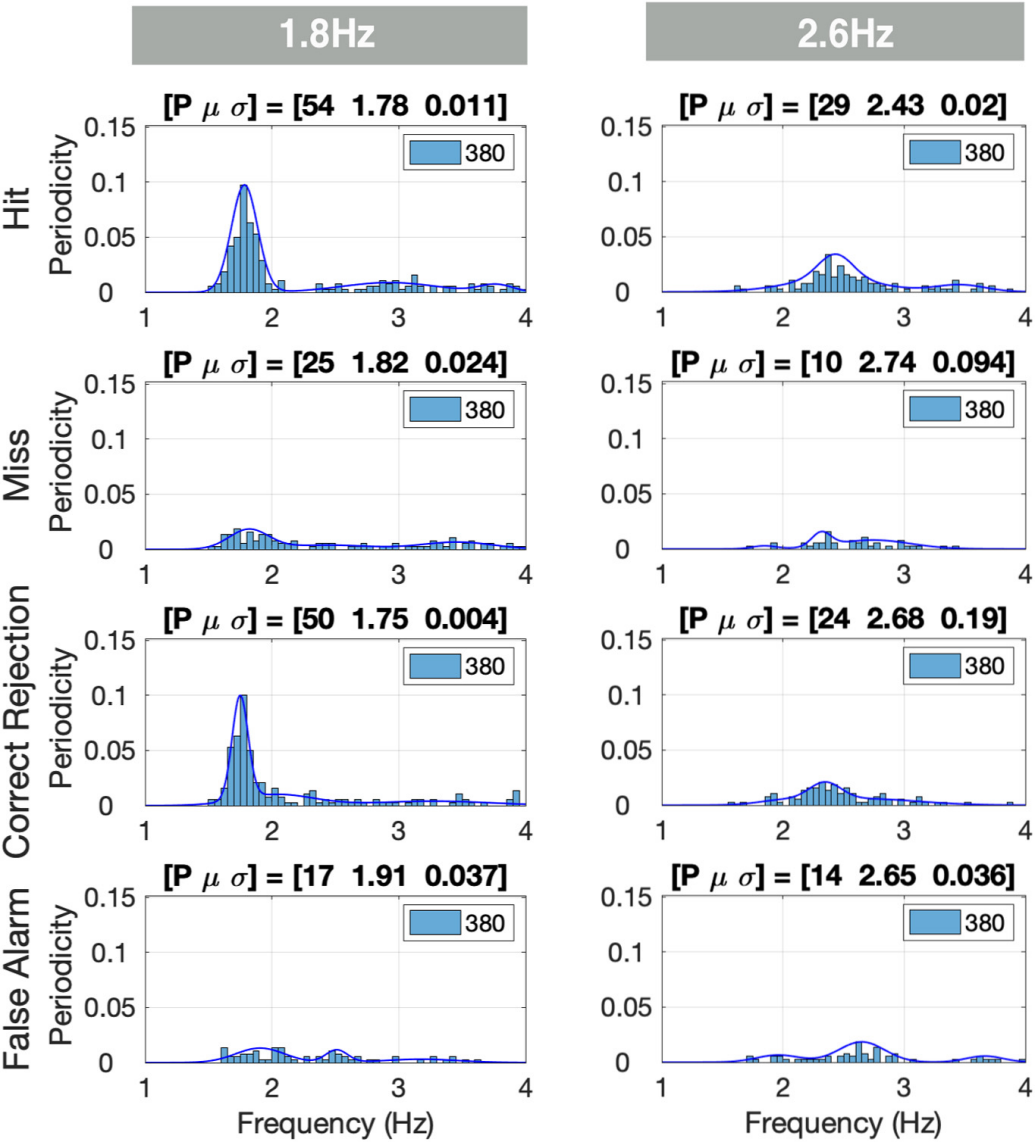
PDFs of δ periodicities per response class. The periodicity density functions (PDFs) are displayed for the left hemisphere STG region of interest (ROI). The number of voxels in this ROI is 20 and the number of participants 19. Per voxel and subject, one XCOV peak inside the δ [1 4] Hz range was selected. The rows indicate the response classes (hit, miss, etc.), and the columns the chunking conditions. Each entry shows the histogram (with the periodicity count scaled to L × N), and the corresponding PDF. The inset of each entry shows the total number of data points L × N analyzed (20 voxels × 19 subjects = 380 incidences). The goodness of the PDF is quantified in terms of the percentage (*p* value) of datapoints inside the frequency range of interest with respect to the total number of datapoints, and the mean μ and variance σ of the prominent Gaussian component of a third order GMM. For the 1.8-Hz condition, a strong periodicity presence at 1.8 Hz was recorded for the hit and correct rejection responses, with P over 50%. A much weaker presence was recorded for the miss and false alarm responses. A similar trend is shown for the 2.6-Hz condition, albeit with much weaker periodicity presence compared with the 1.8-Hz condition, and a smaller number of datapoints (P of below 30%).

We used the XCOV of periodicity across M trials to determine whether the elicited brain signal measured at any given voxel within a specific ROI shows periodicity, and if so, to extract the frequency. Then, we derived the PDF of the periodicities across all voxels in the ROIs of interest (Fig. 2). The goodness of the periodicity is quantified by in terms of P, the percentage of datapoints inside the frequency range of interest with respect to the total number of datapoints (L voxels × N subjects), and the mean μ and variance σ of the prominent Gaussian component of a third order GMM. Figure 5 shows the periodicity PDFs in the [1 4] Hz frequency range for the STG ROI in the left hemisphere. For the 1.8-Hz condition, a strong periodic response at ∼1.8 Hz was recorded for the hits and correct rejections, with the P over 50%. Much weaker presence of periodicity was recorded for the misses and false alarms. A similar trend is shown for the 2.6-Hz condition, albeit with much weaker periodicity compared with the 1.8-Hz condition, and with a smaller P (of below 30%). Notice that, across chunk conditions, the PDF patterns for hits and correct rejections are similar, as are the patterns for misses and false alarms. Such similarities were observed for all ROIs. Therefore, in presenting the rest of the data, the hits and correct rejections are combined to indicate correct responses, and the misses and false alarms are as erroneous responses.

In the following figures, the data are presented as follows. Each figure contains 6 × 2 entries organized in six rows (ROIs) and two columns (chunking conditions). Each entry shows the periodicity PDF, and the goodness of the periodicity is quantified in terms of P, μ and σ. In some selected entries, the upper left corner shows the Kullback–Leibler divergence (KLD) of the entry’s PDF with respect to a reference PDF defined in the respective figure caption. Finally, in some entries, no μ and σ values are present. This is so because of a failure of the third order GMM to converge because of the small *p* value.

**Figure 6.**
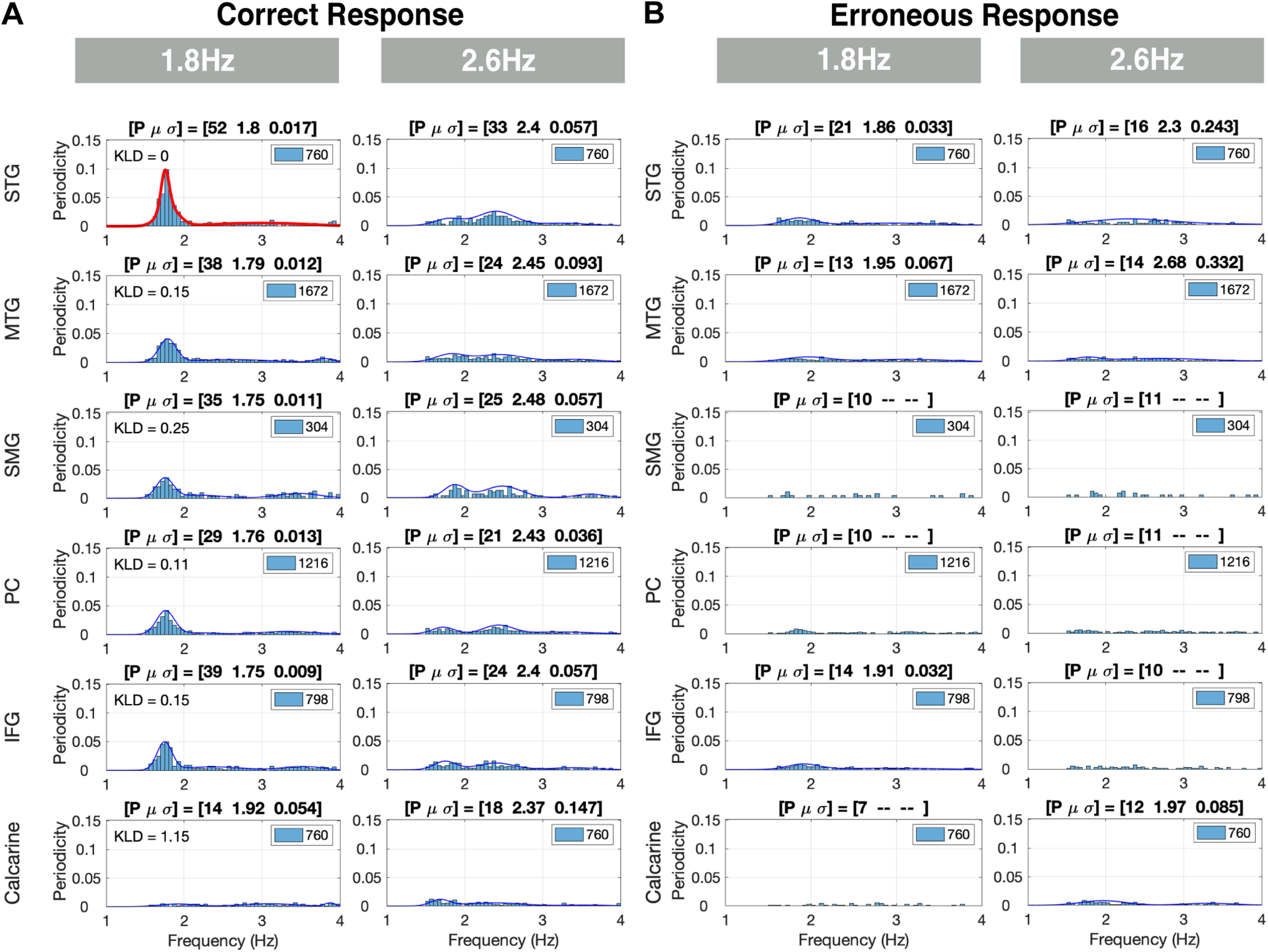
δ Periodicities for correct and erroneous responses in the left hemisphere. ***A***, Periodicities for correct responses: rows indicate the ROIs and columns the chunking conditions. Each entry shows the histogram (with the periodicity count scaled to the L × N), and the PDF, quantified in terms of the percentage (*p* value) of datapoints inside the frequency range of interest with respect to the total number of datapoints (L × N, see inset). For the 1.8-Hz condition, a strong periodicity presence at ∼1.8 Hz is recorded. A similar trend is shown for the 2.6-Hz condition, albeit with much weaker periodicity presence compared with the 1.8-Hz condition. The 1.8-Hz condition column shows the KLD computed for this condition at all ROIs, with respect to the STG ROI highlighted in red (upper left corner of the ROIs). The KLD values suggest similar patterns of elicited δ periodicities in the temporal brain areas (STG and MTG ROIs), and in the frontal motor and temporal parietal areas (IFG, SMG, and PC ROIs). ***B***, Periodicities for erroneous responses: no presence of periodicities is recorded for any condition.

Figure 6*A*,*B* show the elicited responses in the [1 4] Hz frequency band for correct responses (i.e., hits and correct rejections combined), and erroneous responses (i.e., misses and false alarms combined), respectively. We term these elicited responses δ responses. For correct responses in the 1.8-Hz condition a strong periodicity presence at ∼1.8 Hz is recorded. A similar pattern is shown for the 2.6-Hz condition, albeit with much weaker periodicity presence compared with the 1.8-Hz condition (lower *p* value and wider σ). For erroneous responses, for all ROIs, no presence of periodicities is recorded, for any condition. More specifically: for correct responses, in the chunked conditions, the auditory association ROI (STG) shows a compelling periodicity presence at 1.8 Hz in the 1.8-Hz condition and a weaker presence at 2.6 Hz in the 2.6-Hz condition. At the middle temporal ROI (MTG), periodicity exists for the chunked conditions, albeit with 1.8 Hz periodicity stronger than that of 2.6 Hz. Similar patterns are observed in the speech-motor planning and integration ROIs (IFG, SMG, PC), whereas periodicity is present at 1.8 Hz, and is absent in the 2.6-Hz condition. Note that in the visual ROI (calcarine), δ periodicities are absent for all conditions. Finally, the 1.8-Hz condition column of Figure 6*A* also shows the KLD for all ROIs, with respect to the STG ROI (highlighted in red). The KLD values suggest similar patterns of elicited δ periodicities observed in the temporal brain regions (STG and MTG ROIs, with KLD value of 0.15 for MTG), and the frontal motor and temporal-parietal regions (IFG, SMG and PC ROIs, with KLD values of 0.15, 0.25, and 0.11, respectively).

Furthermore, we compared the elicited δ responses in all ROIs in the left versus the right hemispheres for correct responses. Similar periodicity PDFs are observed for all ROIs in all chunking conditions. The KLD was calculated for each ROI in the Right hemisphere against the corresponding left ROI. The KLD values show a closer similarity between the periodicity PDFs of the left and right hemisphere of the temporal brain regions (STG and MTG, with KLD values of 0.1 and 0.11, respectively). In contrast, in the frontal motor and temporal-parietal regions periodicities were more prominent in the left compared with the right hemisphere (IFG, SMG, and PC, with KLD values of 0.28, 0.15, and 0.47, respectively).

**Figure 7.**
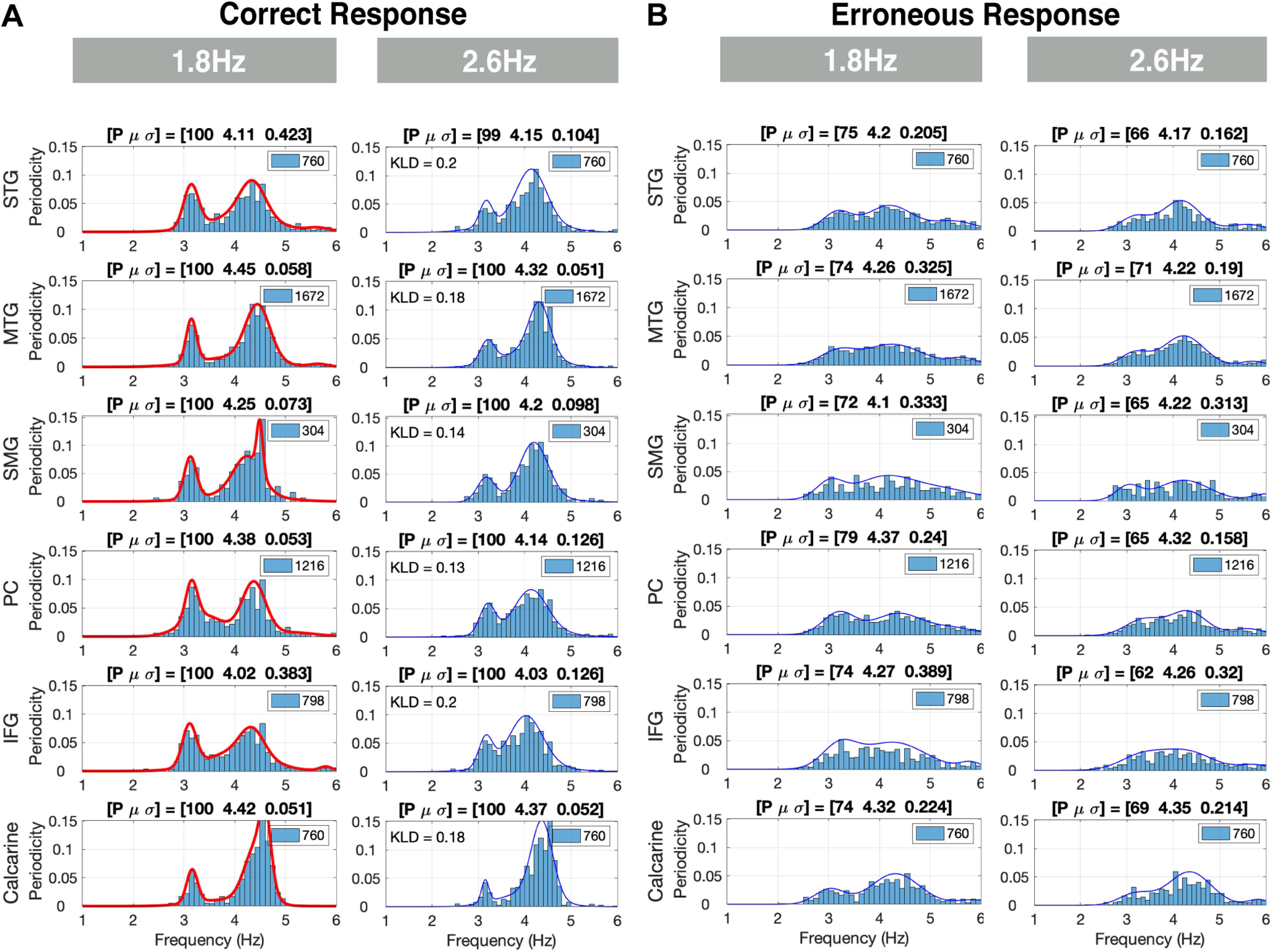
θ Periodicities for correct and erroneous responses in the left hemisphere. ***A***, Periodicities for correct responses: strong θ periodicities were present in all regions of interest (ROIs) and for all chunking conditions. Such elicited neural response patterns reflect the single digit presentation rate. The histograms are scaled to L × N (see inset). The periodicity density functions (PDFs) are similar in shape across conditions, as is quantified by the KLD values comparing the PDFs in the 1.8-Hz condition with respect to the 2.6-Hz condition. ***B***, Periodicities for erroneous responses: a weaker more dispersed presence of θ periodicities is recorded for all conditions (lower *p* value and wider σ).

Figure 7*A*,*B* show the elicited responses in the [2 6] Hz frequency band for the correct and erroneous responses, respectively, for ROIs in the left hemisphere. We term responses in this frequency band θ responses. For the correct behavioral responses, strong θ was elicited in all ROIs and for all chunking conditions. Such elicited neural response patterns reflect the single digit presentation rate. Two observations are noteworthy, the bimodal characteristic of the PDFs for all chunking conditions, in particular for the 1.8-Hz chunking condition, and the strong, unexpected, θ periodicity presence in the calcarine ROI. For the erroneous responses, a weaker more dispersed periodicity presence was observed. Finally, for the correct responses, the periodicity PDFs were similar in shape across conditions, as was quantified by the KLD values comparing the periodicity PDFs in the 1.8-Hz condition with respect to the 2.6-Hz condition (KLD values between 0.13 and 0.2 across ROIs). The similarity of the PDFs across chunking conditions confirms that the decoding time at the digit level was sufficient across conditions.

### Correspondence between behavioral data and electrophysiological data

**Figure 8.**
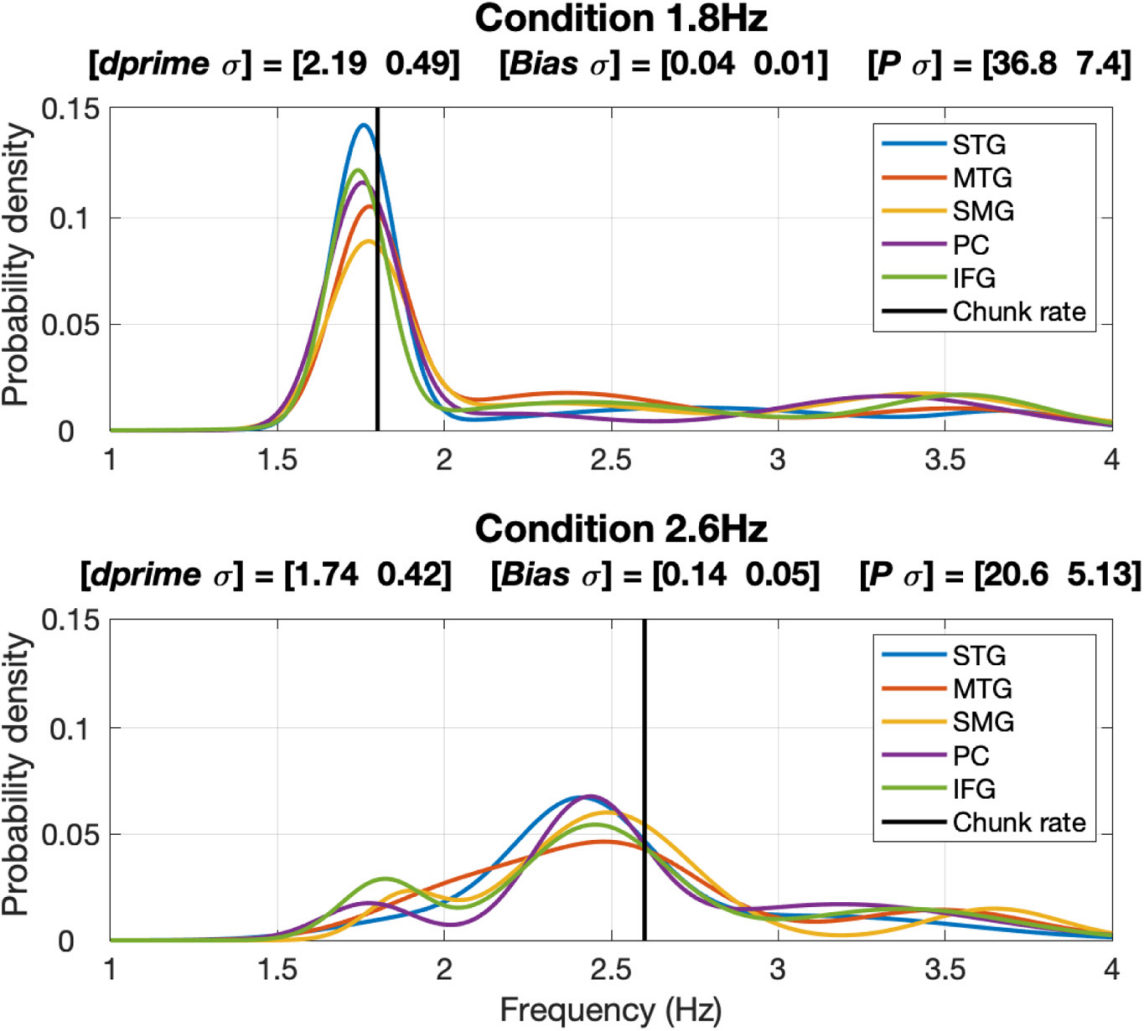
Correspondence between behavioral data and electrophysiological data. Shown are the third order Gaussian Mixture Models (GMMs) of correct responses in the left hemisphere. Unlike Figure 6*A*, which shows the periodicity density function (PDF) scaled to P (the percentage of datapoints inside the frequency range of interest with respect to the total number of datapoints), shown here are the actual probability densities (with the ∫*p(x)dx *=* *1). The title of each panel shows three measures: (1) [*dprime σ*], the behavioral performance; (2) [*Bias σ*], the average of the absolute difference between the mean of the prominent Gaussian component of the GMM and the driving acoustic chunk rate, and variance across the ROIs; and (3) [*p σ*], the average *p* value (defined in Fig. 6) and variance across the regions of interest (ROIs). Note the tightness of the PDFs in the 1.8-Hz condition compared with the pseudo-uniform shape of the PDFs in the 2.6-Hz condition, and the correlation between the decrease in *dprime* and the increase in *Bias* and the decrease in *p*.

Figure 8 quantifies the correspondence between the elicited δ periodicity patterns and the behavioral data. Shown are the third order GMMs computed for the correct responses in the left hemisphere and the two stimulus conditions. Unlike Figure 6*A*, which shows PDF in terms of scaled periodicity count, shown here are the actual probability densities (with the ∫*p(x)dx *=* *1). The title of each panel shows three measures: (1) [dprime σ], the behavioral performance indicated by mean dprime values and the variance across subjects; (2) [Bias σ], the average of the absolute difference (termed Bias) between the mean of the prominent Gaussian component of the GMM and the acoustic chunk rate, and the variance across the ROIs; and (3) [*P* σ], the average *p* value and the variance across the ROIs. Two observations are noteworthy. First, the tightness of the PDFs in the 1.8-Hz condition as reflected in the high probability value at the periodicity frequency, compared with the pseudo-uniform shape of the PDFs in the 2.6-Hz condition. And second, the decrease in dprime accompanies the increase in Bias and the decrease in *P*. These data support the hypothesis that perceptual chunking at the time scale of phrase is derived by acoustic-driven δ oscillators.

## Discussion

In this study we adopted a reductionist approach to test, in electrophysiological terms, the hypothesis that the speech decoding process at the phrasal time scale is guided by a flexible, acoustic-driven neuronal δ oscillator locked to phrase-size acoustic cues (Ghitza, 2017). The proposal suggests an analog role of a δ oscillator, at the phrasal time scale, to the role played by neuronal θ-band oscillations at the syllabic time scale. The study is reductionist in the sense that it is confined to the perceptual chunking of digits sequences, where the digits in the sequence are grouped into phrase-size chunks. We collected, concurrently, behavioral and MEG data during a digit retrieval task, in which the digit sequences were either presented with an acoustic chunk pattern inside or outside of the δ range. Stimuli with a chunk rate inside the δ range elicited considerable neuronal periodicity at the chunk rate in STG, MTG ROIs and IFG, SMG, and PC ROIs. Critically, this pattern of detected periodicities was directly related to correct behavioral responses. In contrast, stimuli with a chunk rate outside of the δ range elicited weak periodicity, aligned with observed declines in behavioral performance. In the calcarine ROI (early visual cortex), considered a “control area” for our analyses, no periodicities at the chunk rate were elicited.

### Presence of δ periodicities in the auditory pathway

How should these activity patterns of neuronal δ and θ periodicities, be interpreted? In the temporal cortex (STG and MTG), robust periodicities were recorded mainly by stimuli with a chunk rate inside the δ range, and only for correct behavioral responses. Periodicities in these brain areas were present even for acoustic chunk rates at the edge of the δ range, albeit considerably weaker. A similar pattern of periodicities was observed in the speech-motor planning and integration areas (IFG, SMG, and PC), where periodicities were absent for acoustic chunk rates outside the δ range. Note that the observed lack of hemispheric lateralization in auditory cortex in our study is in line with previous reports on bilateral θ/δ activity elicited to more complex speech stimuli (Assaneo et al., 2019; Flinker et al., 2019). Interestingly, in contrast to the temporal brain areas, in the speech-motor planning and integration areas more divergence between the left and right hemisphere was observed, with more prominent δ periodicities in the left hemisphere. The left hemisphere more tightly followed the chunking rate compared with the right. These findings suggest an important role for superior and middle temporal and speech-motor planning and integration areas in chunking at the phrasal scale. Importantly, and quite remarkably, the δ-band activity in these areas was fully aligned with behavioral performance (i.e., δ activity was only elicited in correct, but not in erroneous responses). Previously, EEG studies showed δ in bilateral middle and superior temporal areas (also fusiform gyrus; Bonhage et al., 2017) and at frontotemporal sites (Boucher et al., 2019) was related to chunking during phrase and sentence processing. δ Might reflect the chunking of ordered sensorimotor events as articulated sound, rather than syntactic phrasal/sentential processing directly (Boucher et al., 2019). Furthermore, Keitel et al. (2018) and Morillon et al. (2019) recently proposed that δ oscillations in the motor cortex are involved in temporal predictions, affecting speech processing in the auditory cortex at a phrasal scale (for a predictive account of δ see also Breska and Deouell, 2017; Daume et al., 2021; or a statistical learning account, see Henin et al., 2021). A possible interpretation of their findings through the lens of our results is that acoustic-driven segmentation of phrase-size chunks takes place in STG, and the recorded behavioral performance with respect to chunk rate is a consequence of the goodness of segmentation. When the chunk rate is inside the δ band, successful segmentation results in δ activities in speech-motor integration areas (SMG, PC, IFG) that may reflect decoding processes and possibly auditory-motor mapping related processes (Park et al., 2015). In contrast, chunk rates outside of the δ band might result in bad segmentation in STG, and in turn suppressed periodicities in speech-motor integration areas (SMG, PC, IFG) because of unreliable decoding and audio-motor mapping. This interpretation is in line with another study (Donhauser and Baillet, 2020) that reports strong δ activity in STG when the speech input was “informative,” which may be the consequence of appropriate segmentation.

**Figure 9.**
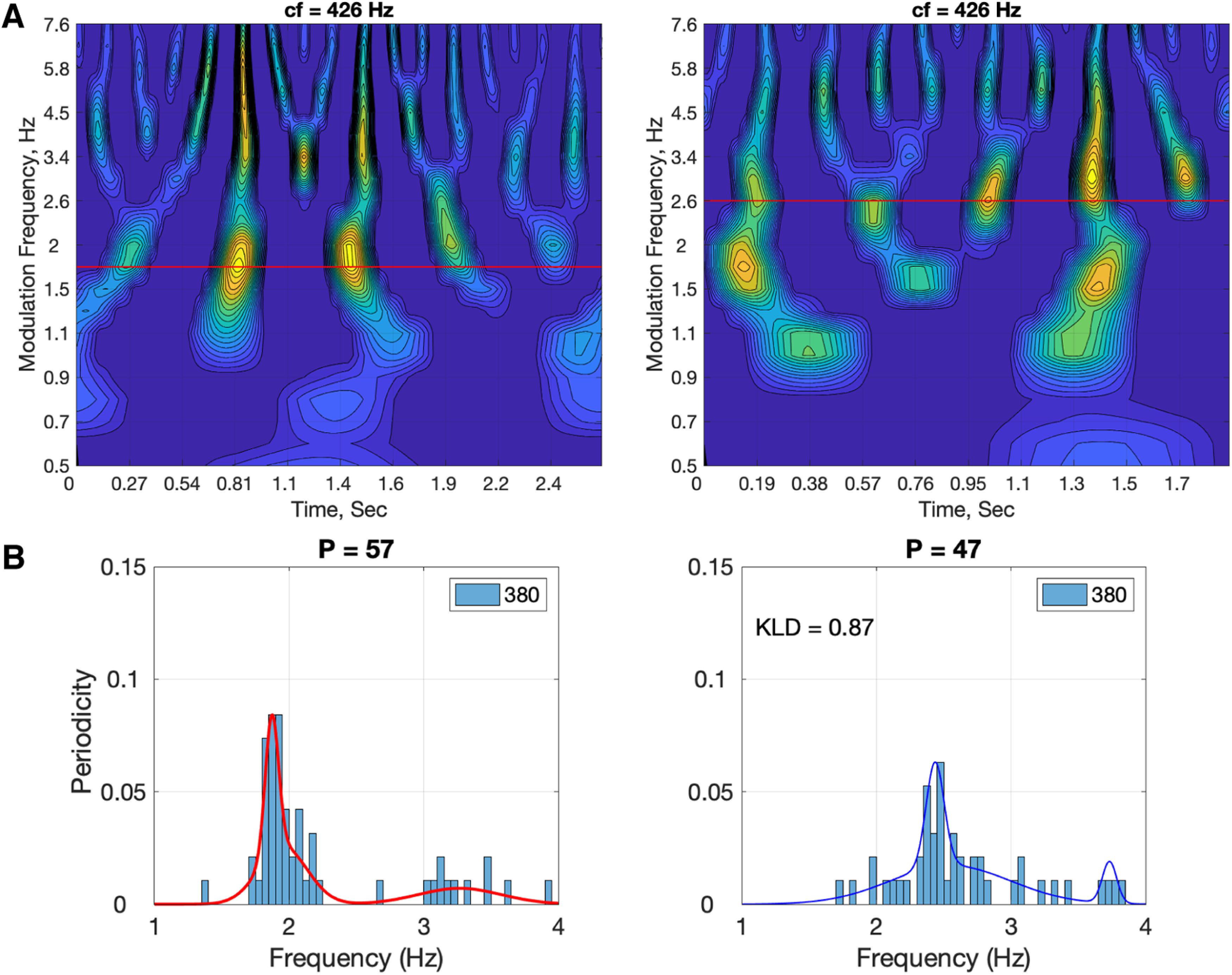
Cochlear modulation spectrum (***A***) and δ periodicities at Heschl’s gyrus (***B***) for the digit-sequence stimuli shown in Figure 1. ***A***, Cochlear output in terms of cochlear modulation spectrum (Jepsen et al., 2008). Shown are modulation spectra of the digit-sequence stimuli shown in Figure 1, for the 1.8-Hz stimulus (left) and for the 2.6-Hz stimulus (right). The modulation spectra shown are snapshots at the cochlear characteristic frequency (CF) of 426 Hz. Abscissae represent time (duration of 2.7 and 1.9 s, for the 1.8- and the 2.6-Hz stimuli, respectively) and the ordinate represents the modulation frequencies (0.5 to 7.6 Hz). Note the strong presence of modulations at 1.8 Hz for the 1.8-Hz stimulus and at 2.6 Hz for the 2.6-Hz stimulus. (***B***) δ Periodicities at Heschl’s gyrus ROI for the correct and erroneous responses, combined. Note that the total number of datapoints is 380: the number of voxels (left and right combined) is 5, the number of participants 19, and the number of response conditions (correct and erroneous) is 4. The KLD value of the 2.6-Hz probability density function (shifted to 1.8 Hz) with respect to the 1.8-Hz probability density function is 0.87. Keeping in mind the concern over the validity of the results because of the limited number of voxels, the strong periodicity presence for both chunking conditions suggest that the diminished periodicity for the 2.6-Hz condition is because of neuronal circuitry characteristics at the STG level and not because of weakening of acoustic cues for chunking.

It could be argued that one cannot draw a conclusive relationship between “chunking” and the neural periodicity in the δ range. In particular, the drop in intelligibility for the 2.6-Hz condition may be because of the fact that the silent gaps in-between the two-digit chunks are shorter. This argument raises three points that merit discussion. First, a question arises whether or not a 2.6 Hz rhythm in the acoustics is present at the cochlear output level. Figure 9*A* shows a simulation of the cochlear modulation spectrum (Jepsen et al., 2008) for a 1.8 Hz (left) and a 2.6 Hz (right) stimuli, taken at a characteristic frequency of 426 Hz (this cochlear place was selected at random, for demonstration). A robust modulation presence is observed for both stimuli, at their respective acoustic input rhythm. Second, it could be argued that the shorter silent gaps result in weaker acoustic cues for chunking. Recalling that neural activity in primary auditory cortex represents sensory representations of the acoustics with a minimal information loss (Nourski et al., 2009), a weakening in acoustic cues should be reflected in terms of a weaker periodicity presence at primary cortex (e.g., the Heschl’s gyrus). As mentioned earlier (Fig. 2), we opted to omit the Heschl’s gyrus from our rigorous periodicity analysis because of the small number of voxels present (three in the left, two in the right). Figure 9*B* shows the XCOV periodicity PDF for all five available voxels, for correct and erroneous responses combined. Keeping in mind the concern over the validity of the results because of the limited number of voxels, we observe a strong periodicity presence for both chunking conditions at their respective chunk rates, suggesting no weakening of the acoustic cues for chunking. In contrast, as early as at the STG level we observe strong periodicities only for chunk rates inside the δ frequency range (Fig. 6). The findings suggest that the neuronal circuitry of the δ oscillator resides at the STG level and constrains prosodic chunking. Third, it could also be argued that the shorter silent gaps result in an insufficient decoding time at the single digit level. However, our data show that this is not the case, as at the digit level, for all chunking conditions and at all ROIs, strong θ periodicities (at the single digit rate) were elicited regardless of the level of behavioral chunking performance. Thus, the drop in performance for the 2.6-Hz condition, with a chunk rate just outside the δ frequency range, is because of the lack of decoding time at the chunk level but not because of digit decoding time. Recall that for both the 2.6-Hz and the 1.8-Hz stimuli, the two-digit chunks themselves have an identical time-compressed acoustics; the only difference is the duration of the silent gaps between the chunks (Fig. 1). Performance is recovered by bringing the chunk rate back inside the δ range, hence providing the extra decoding time needed. As a whole, therefore, our data suggest that segmentation of phrase-sized chunks is realized by neuronal δ oscillators, and that the chunk’s decoding time is determined by δ, in analogy to the role of θ in determining the decoding time at the syllable level (Ghitza, 2014).

### Presence of θ periodicities in all chunking conditions

Our data show strong θ periodicities in all ROIs and for all chunking conditions. Such elicited neural response patterns reflect the single digit presentation rate. A bimodal characteristic of the PDFs is observed for all chunking conditions, but in particular for the 1.8-Hz condition. The bimodality arises from the acoustic properties of the stimuli. Consider, for example, the stimulus shown in Figure 1. Three intradigit durations can be identified: (1) the duration between the onset of the first digit of a chunk and the first digit in the following chunk, which gives rise to the chunking rate; (2) the duration between the onset of the first digit and onset of the second digit in a chunk; and (3) the duration between the onset of the second digit in a chunk and the onset of the first digit in the following chunk. This plurality in intradigit durations give rise to a bimodal duration distribution with a skewness determined by the prescribed chunking rate. The skewness is accentuated, in particular, in our 1.8-Hz stimuli. The bimodal nature in the acoustics drives the elicited neural response seen in our data (Fig. 7*A*).

### Oscillations versus evoked responses

Our data show strong δ cortical periodicities while listening to the 1.8 Hz chunked stimuli. Are these brain waves generated by a neuronal oscillator locked to the acoustic chunk rhythm or do they reflect the evoked response to the corresponding acoustic cues? The answer to this question at the syllabic level has been difficult to determine, because the impulse response of the neuronal circuitry to discrete acoustic cues associated with syllables (e.g., acoustic edges, vocalic nuclei) corresponds, in duration, to the θ-cycle range (about [125 330] ms). Doelling et al. (2019) addressed this conundrum by generating simulated outputs of an oscillator model and of an evoked response model, and comparing the quantitative predictions of phase lag patterns generated by the two models against recorded MEG data. They showed that, compared with the evoked response model, a model that includes oscillatory dynamics better predicted the MEG data. Our data provides additional support for the oscillator interpretation. Can the observed, robust periodic responses to a 1.8 Hz chunked stimulus reflect evoked responses elicited by discrete acoustic cues at the phrase time scale? Indeed, steady-state evoked responses to slow dynamics have been observed in both visual and auditory sensory regions (Capilla et al., 2011; Wang et al., 2011). However, only a model of oscillatory dynamics can explain the fact that neural response at the δ range is only present when the acoustic chunk rate is inside, but is absent for rates outside the δ range.

### Generalizability of the neuronal chunking mechanism

#### Scaling up to real speech

The studies discussed above (Meyer et al., 2017; Bonhage et al., 2017; Keitel et al., 2018; Boucher et al., 2019; Morillon et al., 2019) suggest a presence of δ brain waves in phrasal chunking for continuous speech, beyond the digit retrieval paradigm used here. Extending our results to naturalistic speech has important implications for what would constitute optimally sized acoustic chunks for the sentential decoding, or parsing, process. If the information “bound” within windows of roughly a δ cycle are integrated as phrases (intonation phrases and perhaps structural phrases, depending on the specific relation), it suggests that there are natural patterns of spoken phrase rhythms or phrase durations that are best suited for decoding spoken language, driven by the necessity to match a cortical function. Deploying the experimental analysis approach, we describe here to real speech can elucidate the temporal aspects of spoken language comprehension.

#### Infra-δ chunking rate

As discussed earlier we define the relevant δ range to be between 0.5 and 2 Hz, and chose the 1.8-Hz condition to represent the case where the input chunking rate is inside δ, and the 2.6-Hz condition to represent the outside δ case. The main research question of our study was whether elicited δ cortical oscillations correlate with behavior. In particular, does performance deteriorate if the chunk rate is outside the δ range? We addressed this question by looking at an above-δ chunking rate (2.6 Hz), but we did not look at infra-δ rates (e.g., 0.3 Hz). The reason to skip the effects of infra-δ rates stemmed from the fact that the decay time of sensory memory, ∼2 s long (Cowan, 1984), roughly coincides with the lower bound of the δ-cycle duration. Consequently, the dominant factor at the origin of a possible deterioration in performance may very well be an internal time constraint on processing spoken material (because of echoic memory span) rather than prosodic segmentation.

In conclusion, oscillation-based models of speech perception (Ghitza, 2011; Giraud and Poeppel, 2012; Gross et al., 2013; Martin and Doumas, 2017; Haegens and Zion Golumbic, 2018; Rimmele et al., 2018b; Lakatos et al., 2019) postulate a cortical computational principle by which decoding is performed within a time-varying window structure, synchronized with the input on multiple time scales. The windows are generated by a segmentation process, implemented by a cascade of oscillators. At the prelexical level, the segmentation process is realized by a flexible θ oscillator locked to the input syllabic rhythm, where the θ cycles constitute the syllabic windows. Doelling et al. (2014) provided MEG evidence for the role of θ, showing that intelligibility is correlated with the existence of acoustic-driven θ neuronal oscillations.

Our major finding, that phrase-size chunking of digit strings is correlated with acoustic-driven δ oscillations, suggests that the role played by neuronal θ-band oscillations in syllabic segmentation can be generalized to the phrasal time scale. The segmentation process is realized by a flexible δ oscillator locked to the input phrase-size chunk rhythm, where the δ cycles constitute the phrase-size chunk windows.

Future research is required to investigate whether our findings can be generalized to continuous speech (i.e., beyond digit strings). That is, whether the intonational phrase patterns of language could be constrained by cortical δ oscillations. Adopting the view that the strategy of composing syllables and words into phrasal units is the result of an evolutionary trajectory to match a cortical function (Patel and Iversen, 2014; Bosman and Aboitiz, 2015), we hypothesize that the phrases of language are constrained by δ oscillations, and the rules of chunking in speech production may be the product of common cortical mechanisms on both motor and sensory sides, with δ at the core.

## Synthesis

Reviewing Editor: Satu Palva, University of Helsinki Decisions are customarily a result of the Reviewing Editor and the peer reviewers coming together and discussing their recommendations until a consensus is reached. When revisions are invited, a fact-based synthesis statement explaining their decision and outlining what is needed to prepare a revision will be listed below. The following reviewer(s) agreed to reveal their identity: Lars Meyer, Peter Lakatos.

The authors have well addressed my concerns and I am happy with most of the revisions. I have one remaining important point.

MAJOR

The authors report (27/534ff) that they observed “XCOV periodicity” for both the 1.8 and 2.6 Hz conditions and the new figure seems to suggest so. Still, I am not fully convinced given that:

(a) the figure shows PDFs instead of histograms - what are the absolute numbers of significant cross-correlations in the two conditions? That is, are there potentially less or more in the either of the two conditions?

(b) there is no statistical test that compares the heights of the distributions, so it is unclear whether there is a difference - visually at least, the distribution of the 2.6 Hz condition seems more spread.

(c) the authors show correct trials only in this figure, but shouldn’t the periodicity in the sensory area reflect the periodicity in the stimulus only rather than some high-level decoding related to correct/incorrect? It would be nice to see the data from Heschl’s across all trials (i.e., both correct and incorrect) to the make the argument more consistent with the sensory versus chunking conclusion.

## Author Response

We want to thank the editor and the reviewers for their helpful comments, which are much

appreciated. We have revised our manuscript according to the editor/reviewers’ suggestions, see

our responses below.

MAJOR

The authors report (27/534ff) that they observed “XCOV periodicity” for both the 1.8 and 2.6 Hz

conditions and the new figure seems to suggest so. Still, it is not fully convincing given that:

To address the concerns (a), (b) and (c) below, we modified Fig. 9. In the new figure we show the

scaled histograms (termed periodicity density function, or PDF) and the corresponding GMMs, for

the combined responses (correct and erroneous). In the following, we address the individual

concerns.

(a) the figure shows PDFs instead of histograms - what are the absolute numbers of significant cross-correlations in the two conditions? That is, are there potentially less or more in the either of the two

conditions?

Fig. 9 shows histograms of the XCOV peaks, a peak per datapoint (the total number of datapoints

equals: voxels times subjects, shown in the inset), termed periodicity density functions (PDFs).

Additionally, to the histograms we now show the corresponding GMMs in Fig. 9. Furthermore, we

clarified the figure by changed the naming of the ordinate to make it more intuitive, to

"periodicities” (short for “Periodicities count, scaled to L×N -- the total number of datapoints at the

ROI displayed as inset in the figure panel, see figure caption). The new ordinate name is accordingly

changed in Figs 5-8.

As for the question about the number of significant cross correlations: an XCOV peak is derived by

aggregating the cross correlations that are qualified; the qualification test looks for pairs with “zero

delay” (see line 263). For a typical ROI, the number of cross correlations that pass the test is about

one degree of order bigger than the number of trials (line 268). In line 268 we give the numerical

breakdown for STG, and a similar tradeoff is observed for the Heschl’s Gyrus.

(b) there is no statistical test that compares the heights of the distributions, so it is unclear whether

there is a difference - visually at least, the distribution of the 2.6 Hz condition seems more spread.

In order to quantify the comparison between the conditions we added the KLD value to Fig. 9 and in

the Fig. 9 caption: “The KLD value of the 2.6Hz probability density function (shifted to 1.8Hz) with

respect to the 1.8Hz probability density function is 0.87.”

(c) the authors show correct trials only in this figure, but shouldn’t the periodicity in the sensory area

reflect the periodicity in the stimulus only rather than some high-level decoding related to

correct/incorrect? It would be nice to see the data from Heschl’s across all trials (i.e., both correct

and incorrect) to the make the argument more consistent with the sensory versus chunking

conclusion.

We now analyzed all responses (correct and erroneous; see Fig. 9). The pattern confirms our

previous analysis of only correct responses
